# *Porphyromonas gingivalis* suppresses oral squamous cell carcinoma progression by inhibiting *MUC1* expression and remodeling the tumor microenvironment

**DOI:** 10.1002/1878-0261.13517

**Published:** 2023-09-13

**Authors:** Zhou Lan, Ke‐Long Zou, Hao Cui, Yu‐Yue Zhao, Guang‐Tao Yu

**Affiliations:** ^1^ Stomatological Hospital, School of Stomatology Southern Medical University Guangzhou China

**Keywords:** bacterium therapy, mucin, oral squamous cell carcinoma, *Porphyromonas gingivalis*, tumor microenvironment

## Abstract

Bacteria are the causative agents of various infectious diseases; however, the anti‐tumor effect of some bacterial species has attracted the attention of many scientists. The human oral cavity is inhabited by abundant and diverse bacterial communities and some of these bacterial communities could play a role in tumor suppression. Therefore, it is crucial to find oral bacterial species that show anti‐tumor activity on oral cancers. In the present study, we found that a high abundance of *Porphyromonas gingivalis*, an anaerobic periodontal pathogen, in the tumor microenvironment (TME) was positively associated with the longer survival of patients with oral squamous cell carcinoma (OSCC). An *in vitro* assay confirmed that *P. gingivalis* accelerated the death of OSCC cells by inducing cell cycle arrest at the G2/M phase, thus exerting its anti‐tumor effect. We also found that *P. gingivalis* significantly decreased tumor growth in a 4‐nitroquinoline‐1‐oxide‐induced *in situ* OSCC mouse model. The transcriptomics data demonstrated that *P. gingivalis* suppressed the biosynthesis of mucin *O*‐glycan and other *O*‐glycans, as well as the expression of chemokines. Validation experiments further confirmed the downregulation of mucin‐1 (MUC1) and C‐X‐C motif chemokine 17 (CXCL17) expression by *P. gingivalis* treatment. Flow cytometry analysis showed that *P. gingivalis* successfully reversed the immunosuppressive TME, thereby suppressing OSCC growth. In summary, the findings of the present study indicated that the rational use of *P. gingivalis* could serve as a promising therapeutic strategy for OSCC.

## Graphical abstract

The authors investigated the suppression of oral squamous cell carcinoma (OSCC) growth by *Porphyromonas gingivalis*. The bacterium downregulates MUC1 and CXCL17 expression, which contributes to the reversal of the immunosuppressive tumor microenvironment (TME), leading to OSCC growth inhibition.

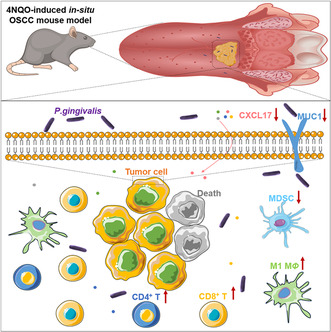

**Abbreviations**
4NQO: 4‐nitroquinoline‐1‐oxide
CCK‐8: cell counting kit‐8
*CXCL17*
: C‐X‐C motif chemokine 17
HE: Hematoxylin–eosin
MDSCs: myeloid‐derived suppressor cells
MOI: multiplicity of infection
*MUC1*
: mucin 1
*MUC2*
: mucin 2
*MUC4*
: mucin 4
*MUC5b*
: mucin 5b
OSCC: oral squamous cell carcinomas
*P. gingivalis*
: *Porphyromonas gingivalis* (*P. g*.)
PDAC: pancreatic cancer
PD‐L1: programmed cell death‐ligand 1
RgpB: Arg‐gingipain
TAMs: tumor‐associated macrophages
TME: tumor microenvironment
Tregs: regulatory T cells

## 1 Introduction

Oral cancer is one of the most common malignant tumors worldwide [1]. Previous studies have shown that some bacterial species can specifically migrate and colonize solid tumors and subsequently activate the host immune response [2]. Accumulating evidence indicates that certain microorganisms and their products, such as *Staphylococcus epidermidis* and *Listeria monocytogenes*, exert antitumor effects [3, 4, 5]. Therefore, the use of bacterial species is thought to be a promising treatment option for oral cancer.

The human oral cavity contains more than 700 species of bacteria, in which *Porphyromonas gingivalis* is reported to be closely associated with the malignant transformation of digestive system tumors [6, 7]. *Porphyromonas gingivalis* was also found to play an essential role in the anti‐oncogenesis effect on oral squamous cell carcinoma (OSCC) by invading the tumor cells and triggering cell cycle arrest at the G1 phase, thereby promoting autophagy of the tumor cells and inhibiting tumor proliferation [8]. In another study, bioengineered *P. gingivalis* encapsulated in an erythrocyte membrane was used together with photothermal therapy and immunotherapy to actively target the tumor microenvironment (TME) and regulate the transformation of M2 macrophages into M1 macrophages for tumor inhibition [9].

As a major component of mucus, mucins are highly glycosylated macromolecular proteins; they are widely distributed in the epithelium of the digestive, respiratory, and genital tracts and are involved in the formation of a mucosal barrier for protecting against external stimuli and mediating intra‐ and extracellular signal transduction [10, 11]. The human genome contains 20 mucin‐encoding genes, including those encoding secretory mucins such as *MUC2* and those encoding membrane‐bound mucins such as *MUC1* [12]. Following their synthesis, mucins undergo two types of glycosylation modification: *N*‐glycosylation and *O*‐glycosylation. *O*‐glycosylated mucins mainly express in epithelial cancer cells, are synthesized by *N*‐acetyl aminotransferases, and are closely associated with the malignant transformation of tumors [13]. GALNT3‐catalyzed aberrant *O*‐glycosylation of mucins in colorectal cancer cells can promote colorectal carcinogenesis by activating the MUC1‐PI3K/AKT signaling axis, which in turn upregulates nuclear factor (NF)‐κB expression [14]. MUC1 and MUC5AC can enhance the proliferative, invasive, and metastatic properties of ovarian and gastric cancers by regulating the E‐cadherin and β‐catenin [15, 16]. The overexpressed mucin can competitively bind to inhibitory receptors on the surface of immune cells such as dendritic cells (DCs), macrophages, and natural killer cells; participate in immune regulation; and mediate immunosuppressive effects [17].

MUC1 can mediate immune escape in triple‐negative breast cancer by promoting the binding of MYC and NF‐κB p65 to PD‐L1 promoter so as to enhance the transcription of programmed death ligand 1 (PD‐L1) [18]. It can also reduce the accumulation of CD8^+^ T cells in tumor tissues through the interferon (IFN)‐γ‐mediated activation of the JAK1‐STAT1‐IRF1 pathway, thereby mediating tumor immunosuppression in breast cancer [19]. MUC1 also binds to Siglec‐9 on the surface of bone marrow cells such as tumor‐associated macrophages (TAMs) and DCs, promotes the secretion of factors related to tumor progression, and activates the MEK–ERK signaling axis to regulate the phenotype of TAMs in the TME to promote tumor progression [20]. In light of these findings, there is abundant potential for tumor therapies targeting *MUC1*. A previous study confirmed that *MUC1* knockdown in glioblastoma cells led to the cell cycle arrest of tumor cells in the G1 phase, thereby inhibiting tumor cell growth [21]. A cancer vaccine targeted at *MUC1* not only prevents the transformation of precancerous tumors into an immunosuppressive microenvironment but also restores the body's immune surveillance against tumors [22].

The TME, which contains a complex cell population as well as extracellular components such as cytokines and chemokines, is considered to promote tumor development [23]. Myeloid‐derived suppressor cells (MDSCs), TAMs, and regulatory T cells (Tregs) can directly or indirectly interact with tumor cells through cytokines and chemokines to restrain the capability of T cells, which in turn mediates tumor development [24, 25, 26]. *CXCL17* is also involved in the intratumoral infiltration of MDSCs and TAMs and thus mediates immunosuppression in tumor tissues [27].

Considering the significance of oral microbiota in OSCC progression, we investigated how *P. gingivalis* regulates the development of OSCC. In the present study, we found that OSCC patients with a higher detection rate of *P. gingivalis* had a better prognosis. An *in vitro* experiment confirmed that *P. gingivalis* inhibited the proliferation of OSCC cells by inducing cell cycle arrest at the G2/M phase arrest and mediating tumor cell apoptosis. The tumor suppressor effect of *P. gingivalis* was further verified in a 4‐nitroquinoline‐1‐oxide (4NQO)‐induced *in situ* OSCC mouse model. Transcriptomics data demonstrated that treatment with *P. gingivalis* downregulated the expression of genes expressing mucins and glycosyltransferases that catalyze the biosynthesis and glycosylation of mucin *O*‐glycan. Histological analyses subsequently confirmed the downregulation of *MUC1* and *CXCL17* expression by *P. gingivalis* treatment. We also performed immunological assays using ELISA and flow cytometry to detect changes in the levels of cytokines and immune cell populations.

## 2 Materials and methods

### 2.1 Sample acquisition and preparation of tissue microarrays

Clinical samples were collected after approved by the Medical Ethics Committee of the Affiliated Stomatological Hospital of Southern Medical University from September 2019 to December 2021, and written informed consent was obtained from each OSCC patient before they went into surgery (Project 2021‐YW‐03‐001). The study methodologies conformed to the standards set by the Declaration of Helsinki. Patients were followed up until the end of the study or death in accordance with the previous study [28]. Hematoxylin–eosin (HE) staining was performed to identify representative tumor areas in OSCC specimens, and tissue cylinders (1.5 mm) were subsequently obtained from the target areas to construct tissue microarrays, including 56 OSCC and 23 paracancerous tissues. For the survival analysis, 11 OSCC patients’ information was lost during follow‐up.

### 2.2 16S rRNA amplicon sequencing and analysis

16S rRNA sequencing was performed by the Biomarker Biotechnology Co. (Beijing, China). The DNA of freshly collected clinical OSCC samples was extracted, and then the V3–V4 regions of the bacterial 16S rDNA genes were amplified. Microbial diversity was analyzed by Paired‐End sequencing on the Illumina Novaseq 6000 platform (Illumina, San Diego, CA, USA). The species composition of the sample was revealed by splicing and filtering the Reads, clustering or denoising, species annotation and abundance analysis. Furthermore, Alpha Diversity analysis, Beta Diversity analysis, significant species difference analysis (LefSe‐LDA Effect Size analysis), correlation analysis, functional prediction analysis (PICRUSt2 function prediction), etc., were performed to explore the differences between samples.

### 2.3 IHC staining

Tissue sections were repaired by sodium citrate under high pressure. *Porphyromonas gingivalis* was detected using polyclonal Rabbit RgpB antibody (1 : 50, Biorbyt, orb51295, Cambridge, UK). Ki67 (1 : 200, CST, 12202T, Boston, MA, USA) was used to show the proliferation of OSCC. MUC1 (1 : 200, Proteintech, 23614‐1‐AP, Wuhan Sanying, Wuhan, Hubei, P.R.C) and Caspase 3 (1 : 200, Proteintecch, 19677‐1‐AP, Wuhan Sanying, Wuhan, Hubei, P.R.C) were used to detect the expression level of MUC1 and Caspase 3. A Leica digital pathology scanner was used to analyze the images. Optical density values were analyzed using aperio imagescope software (Aperio, Vista, CA, USA). Immunohistochemical staining intensity was scored according to four values in excel: Total Intensity of Strong Positive, Total Intensity of Positive, Total Intensity of Weak Positive and Area; the formula is (3 × Total Intensity of Strong Positive) + (2 × Total Intensity of Positive) + (1 × Total Intensity of Weak Positive)/Area (in units per μm^2^) and the resulting value is the average optical density value of the selected area for each sample. Before statistical analysis, the data need to be homogenized, scaled up or down by equal proportions, and checked that the maximum value does not exceed 100 or 300. All procedures could refer to our previous work [28].

### 2.4 Fluorescence *in situ* hybridization (FISH)

FISH was carried out by Sevier Biotechnology (Wuhan, China). Tissue Microarrays were tested with 1 μm *P. gingivalis* 16S rRNA‐specific oligonucleotide POGI 5′‐CY3‐CAATACTCGTATCGCCCGTTATTC‐CY3‐3′. Images were observed and collected under a positive fluorescence microscope (Nikon, Tokyo, Japan). DAPI glows blue at UV excitation wavelength 330–380 nm and emission wavelength 420 nm; CY3 glows red at excitation wavelength 510–560 nm and emission wavelength 590 nm.

### 2.5 Culture of cells

Cell lines CAL27 (RRID: CVCL_1107) were purchased from the American Type Culture Collection (ATCC). SCC7 (RRID: CVCL_V412) was purchased from Ubigene Biosciences (Guang Zhou, China). Their genotypes were confirmed by STR sequences, both mycoplasma‐free. They were cultured in Dulbecco's modified Eagle medium (DMEM, Gibco, California, USA) and RMPI 1640 medium (1640, Gibco, California, USA), 10% FBS (Gibco, California, USA) and 1% penicillin and streptomycin, respectively. SCC7 was supplemented with 5 μg·mL^−1^ of puromycin (Cayman, Michigan, USA). Incubation was at 37 °C and 5% CO_2_.

### 2.6 Culture of *P. gingivalis*

*Porphyromonas gingivalis* ATCC BAA‐308 (W83) was purchased from the Guangdong Microbial Culture Collection Center (GDMCC, Guangzhou, Guangdong, P.R.C). The bacterial medium consisted of brain heart infusion solution (BHI, AOBOX, 02‐348), hemin (1 mg·mL^−1^, AOBOX, 04‐102) and menadione (1 mg·mL^−1^, AOBOX, 04‐103). After overnight incubation in an anaerobic environment, *P. gingivalis* were collected by centrifugation at 7000 *g* per 4 °C, washed once with PBS before use. *P. gingivalis* used for oral coating was resuspended in 2% carboxymethylcellulose.

### 2.7 Cell counting kit‐8 (CCK8) assay

CCK‐8 kit (Dojindo, Japan, CK04) was used to measure proliferation of CAL27 and SCC7. CAL27 1 × 10^4^ and SCC7 cells were placed in 96‐well plates and the cells then infected with different multiplicities of infection (MOI) *P. gingivalis* (MOI = 0, 1, 10, 100, 1000) for 0, 6, 12, 24 h without penicillin and streptomycin. After cultured for 2 h, the OD450 was tested with MD SpectraMAX. Finally, the extreme values at both ends were removed to determine the average. Five replicates were set up for each experiment.

### 2.8 RNA‐Sequencing (RNA‐Seq)

RNA‐Sequencing was performed by NovoTech. We provided cell samples and tumor tissue samples (both including control and *P. gingivalis* groups). CAL27 5 × 10^6^ was inoculated in 6‐well plates and precultured for 12 h and then incubated with or without *P. gingivalis* (MOI = 1000) for 6 h without penicillin and streptomycin. Tumor tissues were excised at the end of the experiment and stored in tissue preservation solution for transport. The extracted samples were quality‐checked for total RNA and libraries were sequenced on an Illumina Novaseq platform, followed by relevant data analysis and packaging of the analysis results files.

### 2.9 Mouse model of OSCC *in situ*

Female C57BL6/J mice (*n* = 14) 6–8 weeks old were purchased and fed in the Experimental Animal Center of Southern Medical University. All mice were fed with 4NQO drinking water (50 μg·mL^−1^, N8141‐5G) to induce OSCC. The drinking water was changed every 5 days for 4 months. After 4 months, the mice were randomly divided into two groups (*n* = 7 per group). Two mice in the control group had died, so every group included five mice. Euthanization was carried out before the tumor was dissected. All experimental operations were approved and performed in accordance with the guidelines of Institutional Animal Care and Use Committee of Southern Medical University (Project SYXK2015‐0150).

### 2.10 Flow cytometry

Flow cytometry was used to detect immune cells populations in tongue, spleen, and draining lymph nodes. First, a single‐cell suspension was prepared in accordance with our previous work [29]. CD45 positive selection kit (STEMCELL, 18945, Vancouver, Canada) was used to collect CD45^+^ immune cells. Subsequently, the cells were stained with flow antibodies as in a previous study [29]. Unstained control, single color compensation control and compensation beads were used for gating strategy. Finally, samples were measured by CytoFLEX flow cytometer (Beckman, Bria, California, USA), and the results were analyzed by flowjo (Tree Star, BD, New Jersey, USA).

### 2.11 ELISA

The CXCL17 assay kit (Wuhan Huamei Biotech Co., Ltd, CSB‐EL006246MO, Wuhan, Hubei, P.R.C) was used to determine the expression of *CXCL17* in tumor tissues, and the operation was carried out strictly according to the assay kit instructions.

### 2.12 Data analysis

Data analyses were performed with graphpad prism version 9.0 (GraphPad Software Inc., La Jolla, CA, USA) and SPSS Version 20.0 (IBM Corp. Armonk, New York, USA) for Windows. Normality and Lognormality tests, unpaired *t*‐test, one‐way ANOVA, two‐way ANOVA and nonparametric tests were carried out to analyze significant differences. All experiments were independently repeated in triplicate. Univariate and multivariate survival analysis were carried out by SPSS Version 20.0. The best cut‐off points of *P. gingivalis* expression were selected according to the previous study [30]. Data are presented as mean ± SD. *P* < 0.05 was considered statistically significant. (*P < 0.05; **P < 0.01; ***P < 0.001; ns, not significant).

## 3 Results

### 3.1 Higher abundance of *P. gingivalis* indicates better prognosis of patients with OSCC

**Fig. 1:**
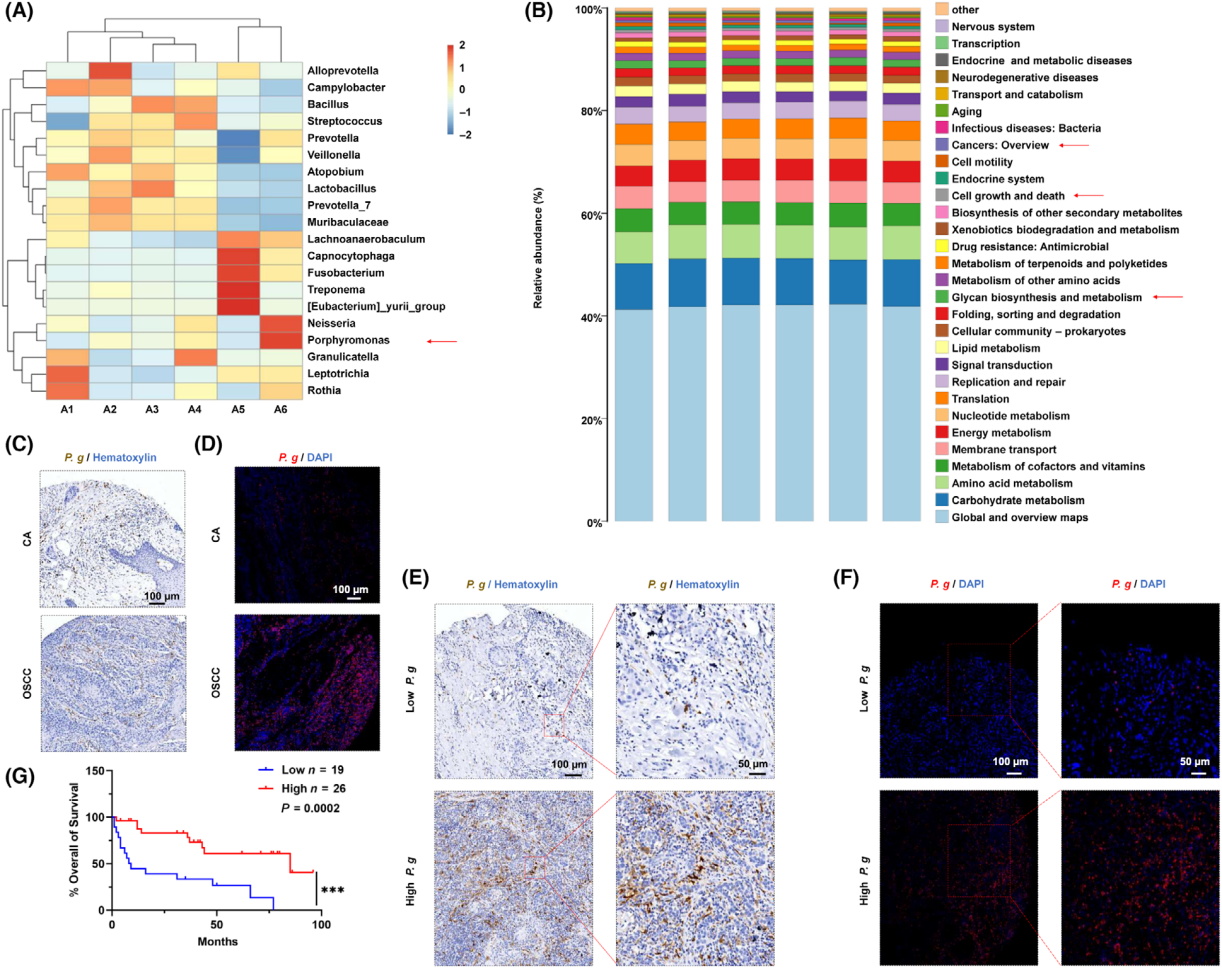
The distribution of *P. gingivalis in* OSCC. (A) Heatmaps of *P. gingivalis* abundance in OSCC, A1–A6 indicate six OSCC tumor tissues. (B) The KEGG functional prediction analysis of microbiomes. (C) Representative IHC pictures of *P. gingivalis* in control and OSCC group using monoclonal mouse anti‐RgpB antibody (brown: *P. gingivalis* colonization; blue: DAPI; CA, adjacent cancer = 23; OSCC, oral squamous cell carcinoma = 56; *P. g*., *P. gingivalis*; scale bar = 100 μm). (D) Representative FISH pictures of *P. gingivalis* in control and OSCC specimens by a Cy3‐labeled *P. gingivalis* 16S rDNA‐directed probe (red: *P. gingivalis* colonization, blue: DAPI; CA = 23, OSCC = 56, scale bar = 100 μm). (E) Representative IHC images of *P. gingivalis* in human OSCC samples (*n* ^low^ = 19, *n* ^High^ = 26, scale bar = 100 and 50 μm). (F) Representative FISH images of *P. gingivalis* in human OSCC samples (*n* ^low^ = 19, *n* ^High^ = 26, scale bar = 100 and 50 μm). (G) The prognosis of OSCC that was associated with the abundance of *P. gingivalis* (survival analysis, ***P < 0.001).

By referring to the methods for studying intratumor microbiota [31], we conducted 16S rRNA sequencing and found high alpha diversity of the oral cavity microbiome as measured by the Shannon, Simpson, Ace, and Chao1 indices (Fig. S1A–E). Shannon exponential, rarefaction, and cumulative relative abundance curves were flattened out, indicating adequate sampling for data analysis (Fig. S1F–H). We observed varying levels of colonization of *P. gingivalis* in OSCC tissues (Fig. 1A). Furthermore, the Kyoto Encyclopedia of Genes and Genomes (KEGG) pathway enrichment analysis for functional gene prediction revealed a correlation between microbiomes and cancers, cell growth and death, and glycan biosynthesis and metabolism (Fig. 1B). The results of immunohistochemistry (IHC; Fig. 1C) and fluorescence *in situ* hybridization (FISH; Fig. 1D) also confirmed the colonization of *P. gingivalis* in OSCC tissues. No significant difference was observed in the abundance of *P. gingivalis* between 56 OSCC samples and 23 adjacent cancer tissue samples (Fig. S2). To determine the clinical significance of *P. gingivalis*, we conducted univariate and multivariate survival analyses of 45 patients (11 patients were lost to follow‐up); results are shown in Table 1. We categorized the IHC grayscale values as low and high expression groups according to the best cut‐off point for *P. gingivalis* expression (Histoscore = 1.74). Representative IHC and FISH images of *P. gingivalis* colonization of the tumor tissues are shown in Fig. 1E and 1F, respectively. A higher abundance of *P. gingivalis* in tumor sites indicated a better prognosis of patients with OSCC (Fig. 1G).

**Table 1:** Univariate and multivariate survival analysis for primary OSCC patients (*n* = 45). OSCC patients were divided into *P. g.* ^*L*ow^ and *P. g.* ^High^ group by the best cut‐off value (Histoscore = 1.74). CI, confidence interval; HR, hazard ratio; *P. g.*, *P. gingivalis*.

Parameters	Univariate survival analysis		Multivariate survival analysis	
	HR (95% CI)	*P*‐value	HR (95% CI)	*P*‐value
Age	1.594 (0.697–3.643)	0.269	0.956 (0.351–2.608)	0.956
Sex	1.664 (0.386–7.164)	0.494	0.898 (0.133–6.063	0.912
Grade II vs. Grade I	0.855 (0.245–2.987)	0.855	0.734 (0.144–3.746)	0.710
Grade III vs. Grade II	3.857 (1.062–14.002)	0.040*	1.901 (0.426–8.489)	0.400
Tumor size				
T2 vs. T1	1.252 (0.275–5.702)	0.771	4.712 (0.612–36.260)	0.137
T3 vs. T1	2.416 (0.484–12.057)	0.282	3.555 (0.426–29.688)	0.241
T4 vs. T1	3.316 (0.590–18.640)	0.174	10.185 (0.891–116.441)	0.062
Node stage				
N1 vs. N0	2.957 (1.134–7.712)	0.027**	3.171 (0.950–10.586)	0.061
N2 vs. N0	6.844 (2.215–21.148)	0.001***	4.353 (1.099–17.237)	0.036*
*P. g* ^High^ vs. *P. g* ^Low^	0.245 (0.102–0.588)	0.002**	0.111 (0.032–0.385)	0.001***

*P < 0.05; **P < 0.01; ***P < 0.001.

### 3.2 *Porphyromonas gingivalis* promotes OSCC cell death by inducing cell cycle arrest at the G2/M phase

**Fig. 2:**
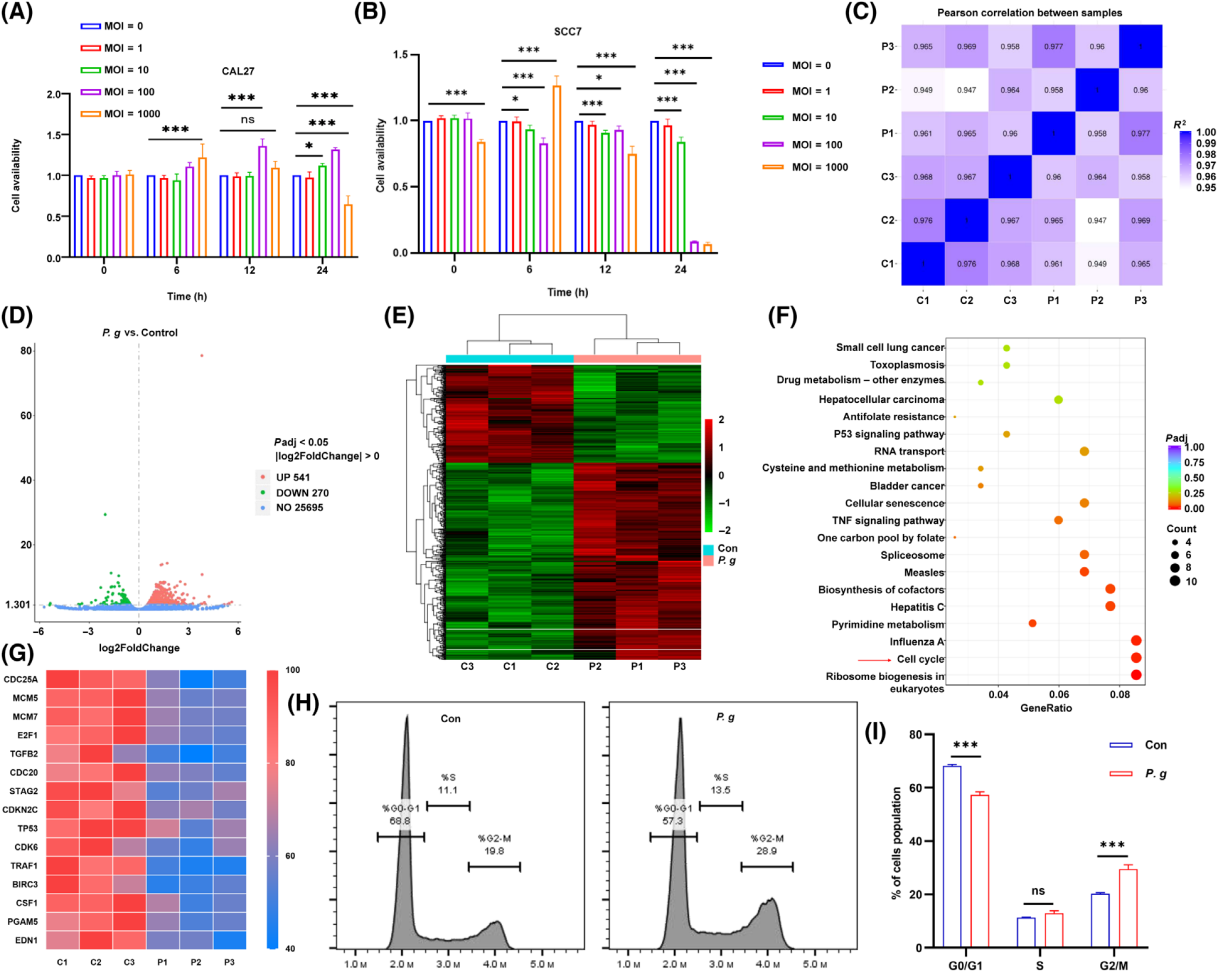
The biological mechanism of *P. gingivalis* treatment in CAL27 by RNA‐Seq. CCK8 assay of cell lines (A) CAL27 and (B) SCC7, treated with *P. gingivalis* at different MOI and times; all data are presented as mean ± SD, *n* = 4 (Two‐way ANOVA with Dunnett's multiple comparisons test, *P < 0.05; ***P < 0.001; ns, not significant). (C) The correlation test in six specimens (*n* = 3). (D) The volcano map of *P. g*. and control group (*n* = 3). (E) The heatmap of significant gene alterations after *P. gingivalis* treatment (*P* < 0.05). (F) KEGG analysis in two groups treated with or without *P. gingivalis* (red arrow indicates *P. gingivalis*‐associated pathways). (G) The individual heatmap of significant downregulated genes refer to cell cycle. (H) Cell cycle related flow cytometry analysis of CAL27 treated with *P. gingivalis* (MOI = 1000) for 6 h (*n* = 3). (I) The distribution of cells in G1, S, G2 phase, the data are presented as mean ± SD, *n* = 3 (Two‐way ANOVA with Sidak's multiple comparisons test, ***P < 0.001; ns, not significant). All experiments were independently repeated in triplicate. (C, Con, control group without *P. gingivalis* treatment; P, *P. g*., *P. gingivalis* treatment group).

Because *P. gingivalis* infection was associated with a better prognosis of OSCC patients, we investigated whether *P. gingivalis* could inhibit OSCC progression. The application of *P. gingivalis* resulted in cell death of CAL27 and SCC7 cells (Fig. 2A,B and Fig. S3). Next, we used RNA sequencing (RNA‐Seq) to determine the potential biological mechanism by which *P. gingivalis* interacts with OSCC cells. CAL27 cells were treated with or without *P. gingivalis* (MOI = 1000). Correlation studies revealed high intra‐group concordance and inter‐group similarity in gene expression between the treated and untreated cells (Fig. 2C). The results of volcano plots and hierarchical cluster analysis showed that 541 genes were upregulated and 270 downregulated (Fig. 2D,E). KEGG pathway enrichment analysis revealed that *P. gingivalis* treatment may be associated with the cell cycle pathway of OSCC cells (Fig. 2F). Hierarchical cluster analysis showed that the cell cycle pathway genes were significantly downregulated in the *P. gingivalis*‐treated group (Fig. 2G). Therefore, we speculated that *P. gingivalis* can exert tumor suppressive effects by affecting the cell cycle to mediate tumor cell death. The 24‐h treatment of *P. gingivalis* could induce cell death (Fig. S4). Subsequent cell cycle detection by flow cytometry analysis confirmed our speculation that tumor cells treated with *P. gingivalis* for 6 h underwent cell cycle arrest at the G2/M phase, accompanied by a decrease in the proportion of cells at the G0/G1 phase and an increase in the proportion of cells at the G2/M phase (Fig. 2H,I).

### 3.3 *Porphyromonas gingivalis* inhibits the growth of 4NQO‐induced OSCC *in situ*

**Fig. 3:**
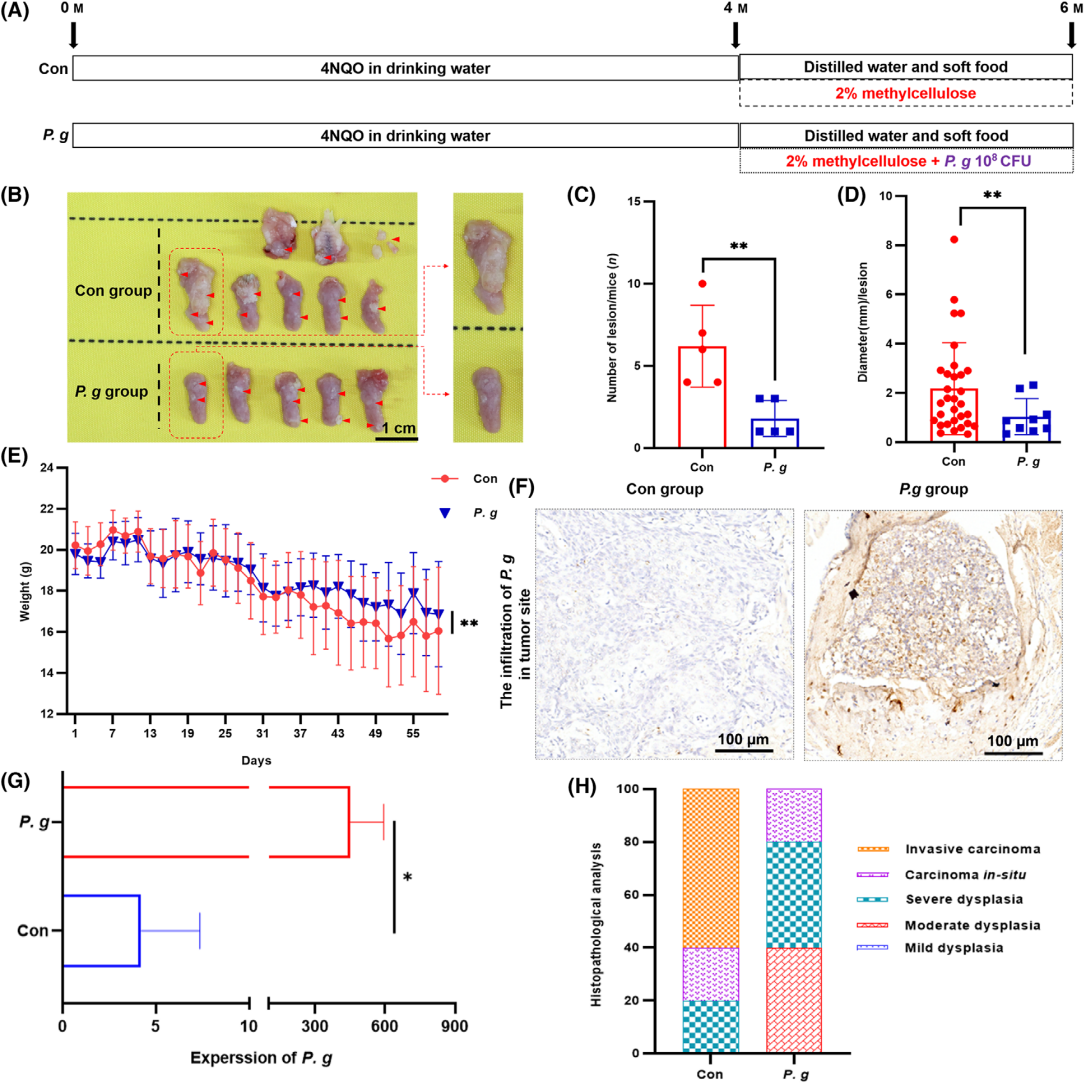
*Porphyromonas gingivalis* inhibits the growth of 4NQO‐induced OSCC *in situ*. (A) Protocol of scheduled animal experiments. (B) Representative pictures of 4NQO‐induced OSCC in con group and *P. gingivalis* group, red circles represent the OSCC tumor tissues (*n* = 5, scale bar = 1 cm). (C) The numbers and (D) diameters of OSCC lesions in con group and *P. gingivalis* group; the data are presented as mean ± SD, *n* = 5 (Mann–Whitney test was used in (C) and unpaired *t*‐test with Welch's correction was used in (D), **P < 0.01). (E) The weights monitoring in con group and *P. g*. group when oral coating began. The data are presented as mean ± SD, *n* = 5 (Unpaired *t*‐test with Welch's correction, **P < 0.01). (F) Representative IHC staining pictures of *P. gingivalis* infiltrated in tumor site (*n* = 3, scale bar = 100 μm). (G) The IHC analysis of *P. gingivalis* expression per μm^2^ in con and *P. gingivalis* group; the data are presented as mean ± SD, *n* = 5 (Unpaired *t*‐test, *P < 0.05). (H) The percentage of OSCC lesion types, including mild to severe dysplasia, carcinoma *in situ* and invasive carcinoma (*n* = 5). All experiments were independently repeated in triplicate. (Con, 2% methylcellulose treatment group; *P. g*., 2% methylcellulose + 10^8^ CFU *P. gingivalis* treatment group).

To further confirm the inhibitory effect of *P. gingivalis* on OSCC development *in vivo*, a C57BL/6J mouse model with *in situ* OSCC was successfully constructed by feeding the mice with 4NQO‐treated water (50 μg·mL^−1^) for 4 months, followed by coating of the oral cavity with or without *P. gingivalis* (1 × 10^8^ CFU) every 2 days for 2 months (Fig. 3A). Based on the images of the oral cavity taken once every month, *P. gingivalis* exhibited a tumor‐suppressive effect (Fig. S5). The mice were euthanized, and photographs of their tongue and tumor site were acquired (Fig. 3B). The *P. gingivalis*‐treated group showed a decreased onset of OSCC, with significantly few tumors of a smaller volume (Fig. 3C,D). Moreover, the *P. gingivalis*‐treated group showed less weight loss than the control group (Fig. 3E). IHC analysis was performed to detect whether *P. gingivalis* successfully infiltrated the 4NQO‐induced tumor sites (Fig. 3F,G) –the results indicated that *P. gingivalis* played an important role in suppressing OSCC progression. Subsequently, HE staining was used to reveal tumor development in the two groups at different stages of tumor progression. The control group showed histopathological manifestations of the tumor, such as severe dysplasia, carcinoma *in situ*, and invasive carcinoma, whereas the *P. gingivalis*‐treated group showed moderate to severe dysplasia and no invasive tumor lesions (Fig. 3H and Fig. S6). These findings suggest that *P. gingivalis* could inhibit OSCC development.

### 3.4 Biological mechanisms of tumor inhibition by *P. gingivalis*

**Fig. 4:**
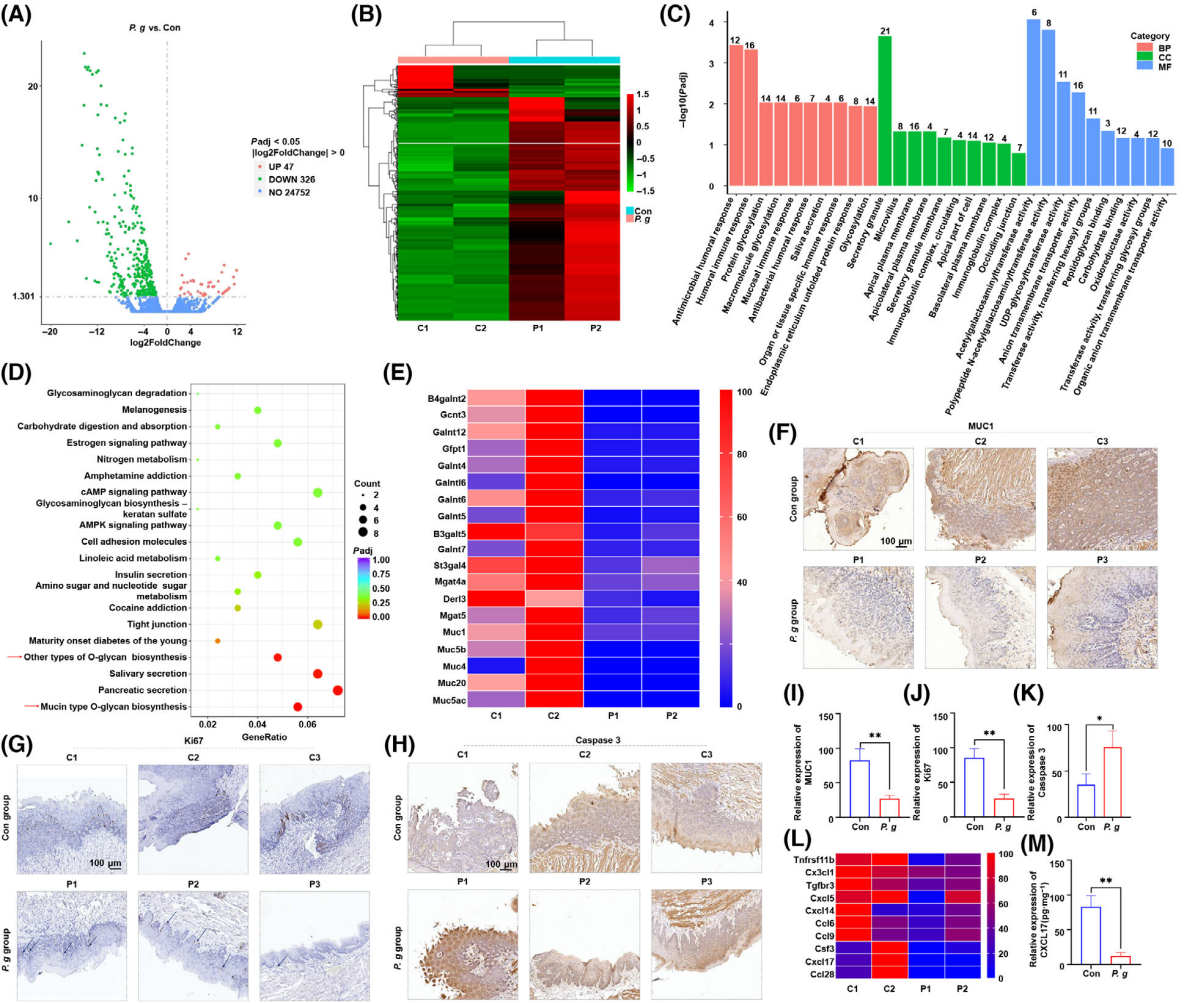
Biological mechanisms of tumor inhibition by *P. gingivalis*. (A) The volcano map of *P. gingivalis* and con group (*n* = 2, *P* < 0.05). (B) The heatmap of significant genes changed after *P. gingivalis* treatment (*n* = 2, *P* < 0.05). (C) The GO analysis of the *P. gingivalis* group vs. con group. ‘BP’, Biological Process; ‘CC’, Cellular Component; ‘MF’, Molecular Function. (D) KEGG pathway enrichment analysis in two groups treated with or without *P. gingivalis* (red arrow indicates *P. gingivalis*‐associated pathways). (E) The heatmap of selected genes related to the above associated signaling pathway. Representative images of (F) *MUC1* and *CXCL17*, (G) Ki‐67, (H) Caspase 3 in con group and *P. gingivalis* group (*n* = 3, scale bar = 100 μm). The expression analysis of (I) *MUC1* and *CXCL17*, (J) Ki‐67, and (K) Caspase 3 in con group and *P. gingivalis* group; all data are presented as mean ± SD, *n* = 3 (Unpaired *t*‐test, *P < 0.05; **P < 0.01). (L) The gene expressions of relative cytokines and chemokines (*n* = 2). (M) The expression analysis of *CXCL17* in con group and *P. gingivalis* group measured by ELISA; the data are presented as mean ± SD, *n* = 3 (Unpaired *t*‐test, **P < 0.01). All experiments were independently repeated in triplicate. (C, Con, 2% methylcellulose treatment group; P, *P. gingivalis*, 2% methylcellulose + 10^8^ CFU *P. gingivalis* treatment group).

Next, we investigated the mechanisms by which *P. gingivalis* inhibits tumor growth. First, RNA‐Seq was performed for tumor tissues. The volcano plots (Fig. 4A) and heatmap (Fig. 4B) revealed that 326 genes were downregulated (*P. gingivalis*‐treated group vs. control group). These differentially expressed genes were subjected to Gene Ontology (GO) analysis. The top 30 enriched terms of the *P. gingivalis*‐treated group and the control group are shown in Fig. 4C. The genes were enriched in terms of regulation of glycosyltransferase activity, glycosylation, and glycoprotein synthesis. KEGG pathway enrichment analysis revealed that *P. gingivalis* treatment significantly inhibited mucin *O*‐glycan synthesis (Fig. 4D), which is related to tumor inhibition [32]. We then focused on the differences in the expression of glycosylation and mucin synthesis‐associated genes. The heatmap of the treated group showed significantly downregulated mucin synthesis‐associated genes (Fig. 4E), particularly *MUC1* and *MUC5b*, which are associated with mucosal immunity [33, 34, 35]. Histological staining also confirmed that *MUC1* expression was significantly decreased in the *P. gingivalis*‐treated group (Fig. 4F,I). We also noted a decrease in the expression level of Ki‐67, which was consistent with the results of the *in vitro* experiment (Fig. 4G,J), suggesting that the proliferation of OSCC cells was inhibited by *P. gingivalis* treatment. Caspase 3 level was significantly upregulated in the *P. gingivalis*‐treated group (Fig. 4H,K), which shows that *P. gingivalis* treatment promotes the apoptosis of OSCC cells.

Pro‐tumorigenic immune cells in the TME play a crucial role in promoting tumorigenesis and tumor progression [35]. Therefore, we examined whether *P. gingivalis* inhibited the recruitment of pro‐tumorigenic immune cells. The heatmap constructed based on the results of transcriptome sequencing of tumor tissues demonstrated that *P. gingivalis* treatment reduced the expression of pro‐tumorigenic chemokines such as *CXCL17* (Fig. 4L), which plays a role in the recruitment of MDSCs and TAMs [36]. The results of ELISA showed that *P. gingivalis* treatment reduced *CXCL17* expression in the TME of the treated group as compared with that in the control group (Fig. 4M). These findings indicate that treatment with *P. gingivalis* alters the expression of *MUC1* and *CXCL17* in tumor tissues, leading to the inhibition of tumor growth in the 4NQO‐induced *in situ* OSCC mouse model.

### 3.5 *Porphyromonas gingivalis* treatment reverses the immunosuppressive TME in the 4NQO‐induced *in situ* OSCC mouse model

**Fig. 5:**
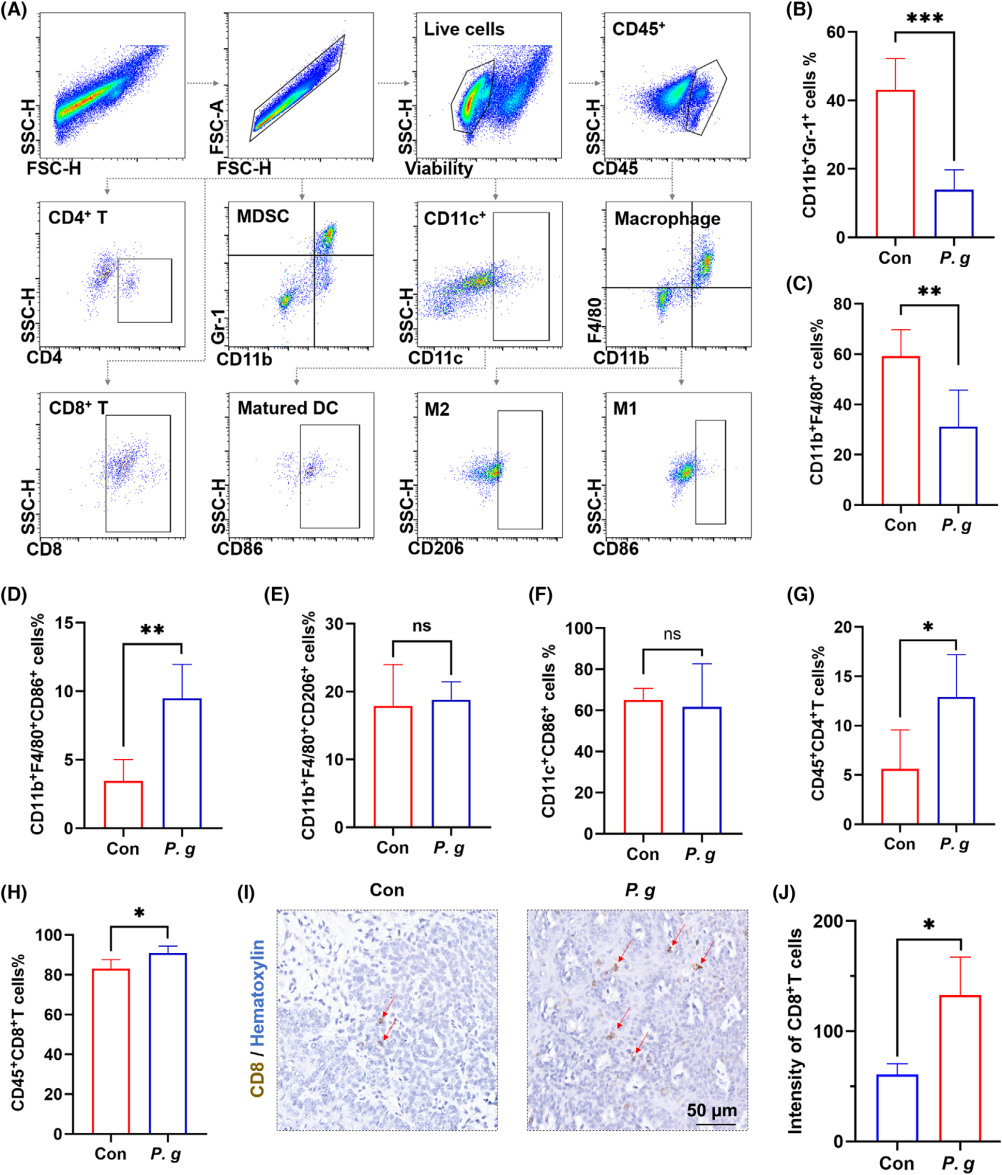
*Porphyromonas gingivalis* treatment reverses the immunosuppressive TME in the 4NQO‐induced *in situ* OSCC mouse model. (A) The gating strategy for different immune cell subsets in TME of 4NQO‐induced mice (*n* = 3 per group). The percentage of tumor‐infiltrating (B) CD11b^+^ Gr‐1^+^ MDSCs (Unpaired *t* test, ***P < 0.001), (C) CD11b^+^ F4/80^+^ Macrophages (Unpaired *t* test, **P < 0.01), (D) CD11b^+^ F4/80^+^ CD86^+^ M1‐like macrophages (Unpaired *t*‐test, **P < 0.01), (E) CD11b^+^ F4/80^+^ CD206^+^ M2‐like macrophages (Unpaired *t*‐test, ns, not significant), (F) CD11c^+^ CD86^+^ matured DCs (Unpaired *t*‐test with Welch's correction; ns, not significant), (G) CD4^+^ T cells (Unpaired *t*‐test, *P < 0.05) and (H) CD8^+^ T cells (Unpaired *t*‐test, *P < 0.05) were compared; all data are presented as mean ± SD (*n* = 3). (I) Representative images of CD8^+^ T cells and (J) IHC analysis of CD8^+^ T cells (Unpaired *t*‐test, *P < 0.05) in con group and *P. gingivalis* group; red arrow indicates CD8^+^ T cells; the data are presented as mean ± SD, *n* = 3, scale bar = 50 μm. All experiments were independently repeated in triplicate. (Con: 2% methylcellulose treatment group; *P. gingivalis*: 2% methylcellulose + 10^8^ CFU *P. gingivalis* treatment group.

To determine how *P. gingivalis* regulates the tumor immune microenvironment, we sorted CD45^+^ immune cells from the OSCC tissues. Fig. 5A shows the gating strategy of different immune cell subsets. The infiltration of MDSCs in the tumor tissues was significantly reduced in the *P. gingivalis*‐treated group (Fig. 5B). Furthermore, the total number of macrophages was decreased in the *P. gingivalis*‐treated group (Fig. 5C). However, the number of CD86^+^ M1‐like macrophages was slightly increased in the *P. gingivalis*‐treated group (Fig. 5D), and no significant difference in the number of CD206^+^ M2‐like macrophages was observed between the two groups (Fig. 5E). Furthermore, both groups showed no significant difference in the number of matured DCs (Fig. 5F). The proportion of infiltrated CD4^+^ T cells and CD8^+^ T cells was significantly elevated in the *P. gingivalis*‐treated group (Fig. 5G,H). To confirm further the changes in the population of effector T cells stimulated by *P. gingivalis* treatment, we compared the degree of CD8^+^ T cell infiltration in both groups by IHC staining; the results showed a higher accumulation of CD8^+^ T cells in the *P. gingivalis*‐treated group (Fig. 5I,J). These immune cell subpopulations partially showed the same trend of variation in lymph nodes and spleens between both groups; however, the differences were not statistically significant (Figs S7 and S8). Taken together, these findings suggest that *P. gingivalis* treatment reverses the immunosuppressive TME to inhibit OSCC progression.

## 4 Discussion

Currently, the relationship between microorganisms and tumors has become a hot topic of research, as certain bacterial species such as *P. gingivalis*, a causative agent of periodontitis [37], have been found to have a regulatory effect on tumorigenesis and tumor progression. Most researchers believe that *P. gingivalis* accelerates the cell cycle of cancer cells, promotes their proliferation, and inhibits their apoptosis [6, 38, 39, 40]. In contrast, a previous study reported that *P. gingivalis* suppressed the proliferation of OSCC cells by inducing cell cycle arrest at the G1 phase, but had no effect on cell apoptosis [8]. Another study found that the encapsulation of *P. gingivalis* in an erythrocyte membrane promoted its colonization of tumor sites, which subsequently induced the phenotypic transformation from M2 macrophages to M1 macrophages to inhibit tumor growth [9]. In the present study, based on tissue microarray and prognostic analysis, we showed that the infiltration of tumor sites by *P. gingivalis* improved the prognosis of patients with OSCC. Additionally, *P. gingivalis* downregulated the expression of the cell cycle signaling pathway‐related genes in the CAL27 cells. The results of the CCK8 assay and flow cytometry analysis further confirmed that *P. gingivalis* suppressed the proliferation of OSCC cells. These findings were corroborated by *in vivo* experiments, which revealed that *P. gingivalis* delayed tumorigenesis and tumor progression of OSCC, indicating the tumor suppressive effect of *P. gingivalis*.

It is known that mucins function as a barrier between the normal epithelium and external microorganisms and thus reduce the contact of immune cells in the subepithelial layer with microorganisms [41, 42]. However, the innate attributes of mucins to defend and repair epithelial cells are hijacked during carcinogenesis, and a high expression of mucins in the malignant cells could enhance intercellular and cell–matrix interactions as well as cell‐autonomous signaling to promote tumor development [43]. For example, *MUC1* and *MUC5AC* affect E‐cadherin and β‐catenin complexes, thereby promoting the proliferation, invasion, and metastasis of ovarian and gastric cancer cells [15, 16]; in contrast, *MUC4* gene deletion in pancreatic cancer cells inhibits tumor progression by regulating apoptosis and cell cycle [32]. In the present study, RNA‐Seq revealed that the mucin *O*‐glycan biosynthesis pathway was significantly downregulated in a 4NQO‐induced *in situ* OSCC mouse model treated with *P. gingivalis*. Since mucin can resist the microbial invasion of epithelial cells in normal tissues, *P. gingivalis* can better invade host epithelial cells when mucin expression is significantly downregulated. The anaerobic and immunosuppressive properties of the TME can further support microorganisms to colonize the tumor site [31]. In the present study, the infiltration of *P. gingivalis* in the tumor site was confirmed by IHC. *MUC1* and *MUC4*, which were highly expressed on the surface of tumor cells, were also downregulated in the *P. gingivalis*‐treated group. Previous studies have shown that the high expression of MUC1 in tumor tissues is closely associated with the poor prognosis of cancer patients, and the high MUC1 expression can promote tumor progression by affecting the intrinsic and extrinsic apoptotic pathways [44]. In the present study, IHC staining showed that *P. gingivalis* treatment inhibited MUC1 expression and promoted caspase 3 expression, indicating an increase in tumor cell apoptosis.

Once microorganisms disrupt the protective barrier, the anaerobic and immunosuppressive properties of the TME further support their colonization of the tumor site [31]. Furthermore, pro‐tumorigenic immune cells in the TME, such as MDSCs, TAMs, and Tregs, exert tumor‐promoting effects following the secretion of a series of chemokines [36]. Our sequencing results showed that the expression of *Tnfrsf11b, CXCL5, CCL6, CCL9, CSF3, CXCL17*, and *CCL28* was significantly downregulated by *P. gingivalis* treatment. Tnfrsf11b can activate the Wnt/β‐catenin signaling pathway to promote gastric cancer progression [45]. CXCL5 can promote colon cancer metastasis by activating the ERK/Elk‐1/Snail and AKT/GSK3β/β‐catenin signaling pathways [46]. In NFE2L2‐mutated head and neck squamous cell carcinoma, *CSF3* recruits MDSCs to mediate chemotherapy resistance [47]. The activation of the β‐catenin‐CCL28‐Tregs signaling axis is also closely associated with the immunosuppressive TME in gastric cancer [48]. The role of CXCL17 in tumor development is controversial. At present, it is believed that CXCL17 has a dual role. A review by Shabaana revealed that CXCL17 is highly expressed in various tumor tissues and is closely associated with tumor cell proliferation, apoptosis resistance, angiogenesis, and metastasis [49]. Precise mechanistic studies have shown that CXCL17 is associated with increased intratumoral infiltration of MDSCs, TAMs, and Tregs that mediate tumor immunosuppression, while decreasing CD4^+^ and CD8^+^ T cells with antitumor properties [27, 49]. However, in the present study, the results of heatmap and ELISA revealed that *P. gingivalis* treatment markedly downregulated CXCL17 expression. This conclusion was also supported by the finding that the number of MDSCs in the TME was significantly decreased in the *P. gingivalis*‐treated group. This finding may be attributed to the regulatory effect of the MUC family proteins on immune cells through CXCL17 [17]. Current studies have shown that MUC1‐ST binds to Siglec‐9 expressed in myeloid cells, which subsequently promotes the secretion of cytokines and chemokines such as CXCL5, CCL2, interleukin (IL)‐6, and IL‐8, and recruit monocytes and neutrophils to induce angiogenesis and extracellular matrix degradation, leading to remodeling of the TME [20]. In the present study, the downregulation of MUC1 expression was accompanied by a decrease in the number of MDSCs in the TME. MUC1‐C can enhance the transcriptional expression of PD‐L1 to mediate immune evasion, while the blockade of the MUC1‐C subunit can effectively enhance the infiltration of CD8^+^ T cells in tumors [18, 19]. In the present study, the number of tumor‐infiltrating CD8^+^ T cells was significantly increased in the *P. gingivalis*‐treated group. In recent studies, some bioengineered live bacteria have been developed for tumor therapy [5, 9, 50]. Given our finding that the high abundance of *P. gingivalis* was positively associated with good prognosis of OSCC patients, bioengineered *P. gingivalis* could be developed as a beneficial antitumor strategy for OSCC therapy.

## 5 Conclusions

**Fig. 6:**
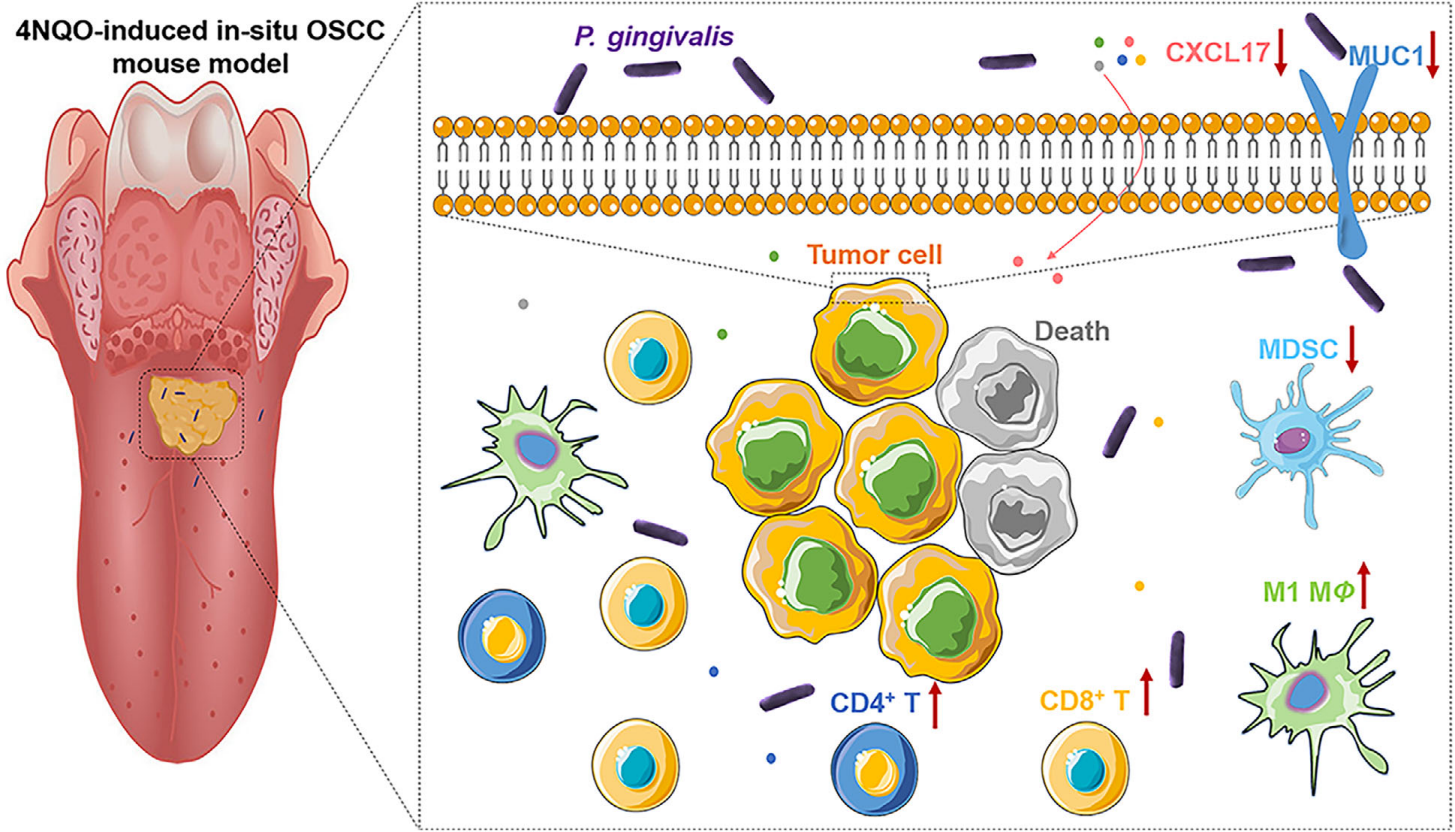
Overview of *Porphyromonas gingivalis* suppression of oral squamous cell carcinoma (OSCC) growth. *Porphyromonas gingivalis* downregulates MUC1 and CXCL17 expression, which contributes to the reversal of the immunosuppressive tumor microenvironment (TME), leading to OSCC growth inhibition.

In summary, we showed that *P. gingivalis* significantly suppressed the growth of OSCC in both *in vitro* and *in vivo* experiments. Although the precise mechanisms by which *P. gingivalis* regulates OSCC progression require further studies, our current results showed that *P. gingivalis* downregulated the biosynthesis of MUC1 as well as the expression of CXCL17, which recruit pro‐tumorigenic immune cells in the TME (Fig. 6). The immunosuppressive TME was reversed by *P. gingivalis* treatment. We also found that a high abundance of *P. gingivalis* was positively correlated with the longer survival of patients with OSCC; this suggests that the use of *P. gingivalis* for tumor suppression could be considered a promising strategy for treating patients with OSCC.

## Conflict of interest

The authors declare no conflict of interest.

## Author contributions

ZL was responsible for conceptualization, data curation, formal analysis, investigation, methodology, validation, writing original draft and reviewing. HC and K‐LZ were responsible for data curation, formal analysis, investigation and reviewing. Y‐YZ and G‐TY were responsible for funding acquisition, supervision, and reviewing.

### Peer review

The peer review history for this article is available at https://www.webofscience.com/api/gateway/wos/peer‐review/10.1002/1878‐0261.13517.

## Supporting information


**Fig. S1.** Alpha diversity of microbiomes in OSCC tissues.
**Fig. S2.** The IHC analysis of *P. gingivalis* expression per μm^2^ in OSCC and CA.
**Fig. S3.** CAL27 and SCC7 treated with *P. gingivalis* at different concentrations and times under microscope.
**Fig. S4.** Flow cytometry analysis of the death ratio of CAL27 and SCC7 disposed with or without *P. gingivalis* (MOI = 1000) for 24 h.
**Fig. S5.** Real‐time monitoring tumors of 4NQO mice disposed with or without *P. gingivalis* for 1 and 2 months (*n* = 7).
**Fig. S6.** Representative pictures of HE staining, containing mild to severe dysplasia, carcinoma *in situ* and invasive carcinoma.
**Fig. S7.** Various infiltrating immune cell subsets in the lymph nodes of 4NQO‐induced mice.
**Fig. S8.** Various infiltrating immune cell subsets in the spleen of 4NQO‐induced mice.

## Data Availability

The data supporting the findings of this study are available from the corresponding author upon reasonable request.
