# Supplementary material for: Porphyromonas gingivalis suppresses oral squamous cell carcinoma progression by inhibiting MUC1 expression and remodeling the tumor microenvironment
Source: Mol Oncol. 2023 Sep 13;18(5):1174–88. doi: 10.1002/1878-0261.13517 (PMC11076995; doi:10.1002/1878-0261.13517)
Supplement: Supplementary file 1 — Fig. S1. Alpha diversity of microbiomes in OSCC tissues. Fig. S2. The IHC analysis of P. gingivalis expression per μm2 in OSCC and CA. Fig. S3. CAL27 and SCC7 treated with P. gingivalis at different concentrations and times under microscope. Fig. S4. Flow cytometry analysis of the death ratio of CAL27 and SCC7 disposed with or without P. gingivalis (MOI = 1000) for 24 h. Fig. S5. Real‐time monitoring tumors of 4NQO mice disposed with or without P. gingivalis for 1 and 2 months (n = 7). Fig. S6. Representative pictures of HE staining, containing mild to severe dysplasia, carcinoma in situ and invasive carcinoma. Fig. S7. Various infiltrating immune cell subsets in the lymph nodes of 4NQO‐induced mice. Fig. S8. Various infiltrating immune cell subsets in the spleen of 4NQO‐induced mice. [file MOL2-18-1174-s001.pdf]

# Supplemental Material

## *Porphyromonas gingivalis* suppresses oral squamous cell carcinoma progression by inhibiting MUC1 and remodeling tumor microenvironment

**Fig. S1**

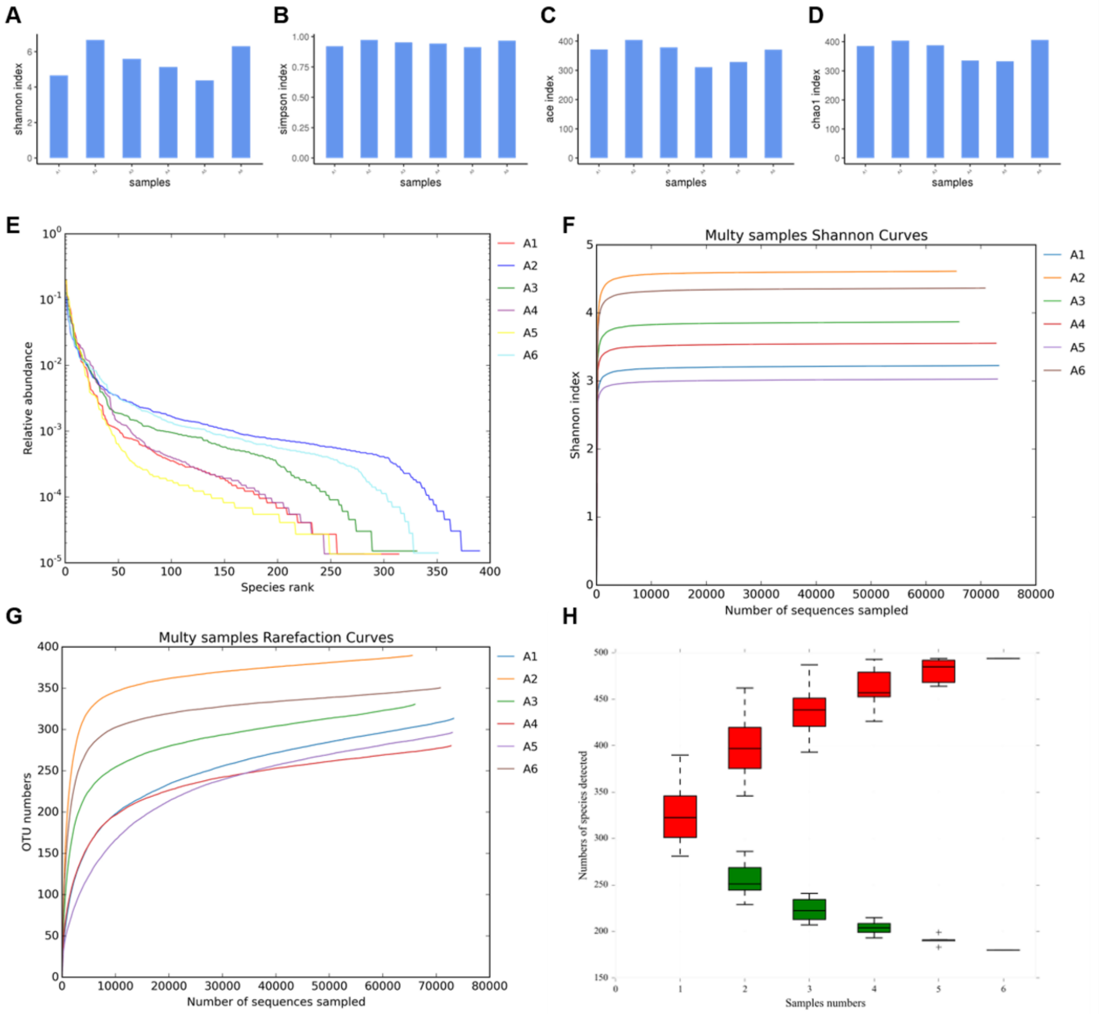


**Fig. S1.** Alpha diversity of microbiomes in OSCC tissues. A) Shannon and B) Simpson indices were used to demonstrate species diversity, the C) Ace indices and D) chao1 indices reflected species richness. E) Rank abundance curve. F) Shannon exponential curve, G) Rarefaction Curve and H) cumulative relative abundance curve of OSCC samples.

**Fig. S2**

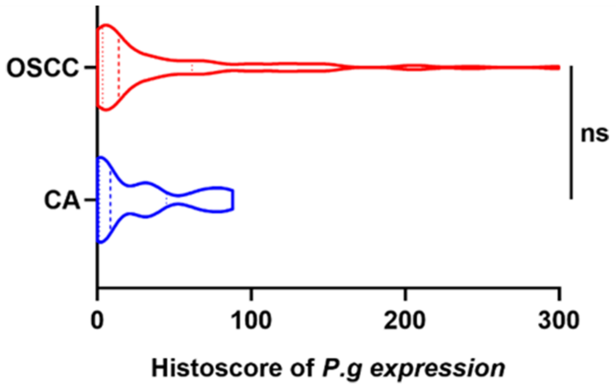


**Fig. S2** The IHC analysis of *P.gingivalis* expression per μm$^2$ in OSCC and CA. CA, Adjacent cancer. The data are represented as Mean±SD, CA=23, OSCC=56 (Mann-Whitney test was used. ns, not significant, scale bar=100μm).

Fig. S3

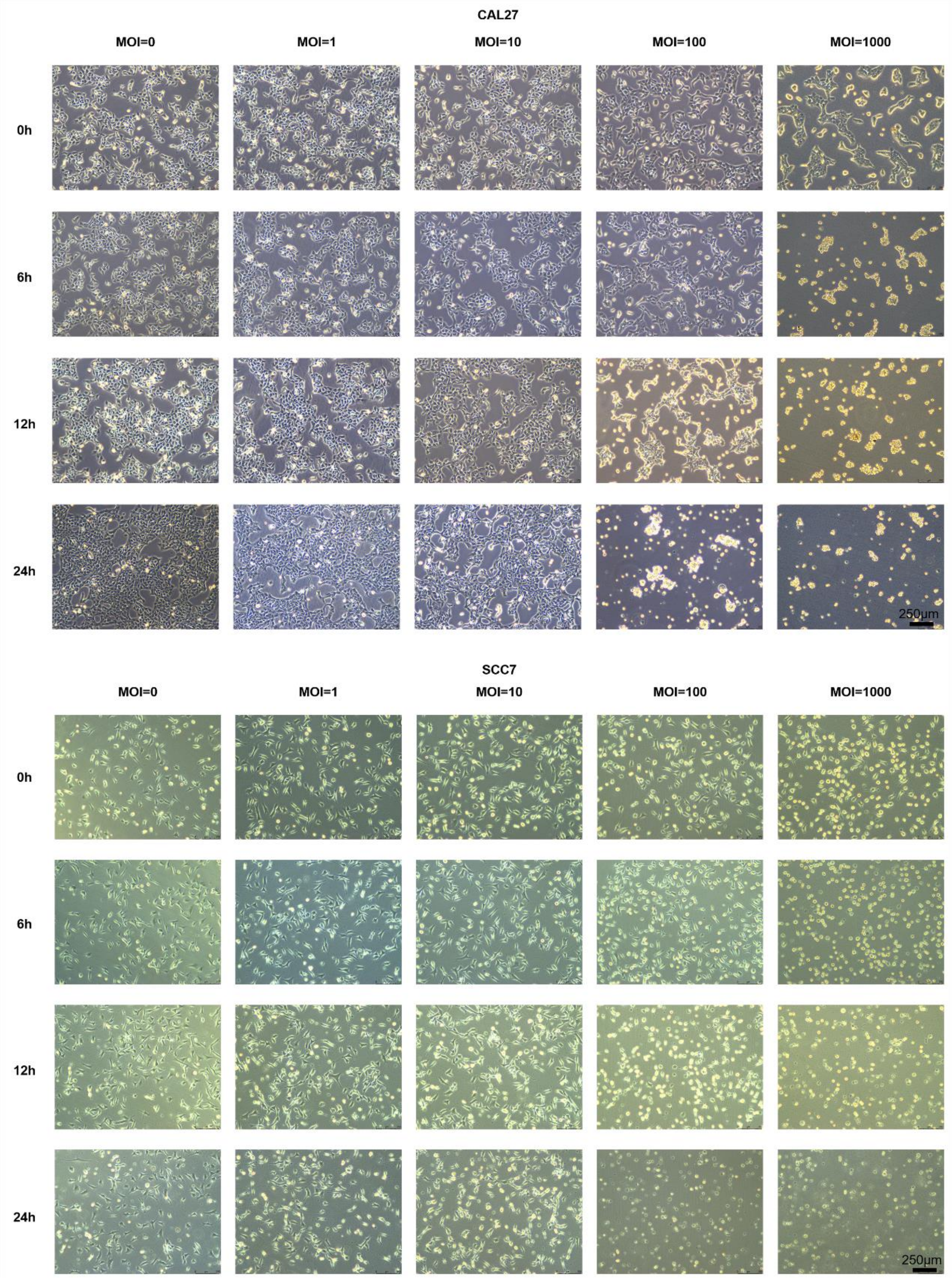


**Fig. S3.** CAL27 and SCC7 treated with *P. gingivalis* at different concentrations and times under microscope. Scale bar = 250 μm. All experiments were independently repeated in triplicate.

**Fig. S4**

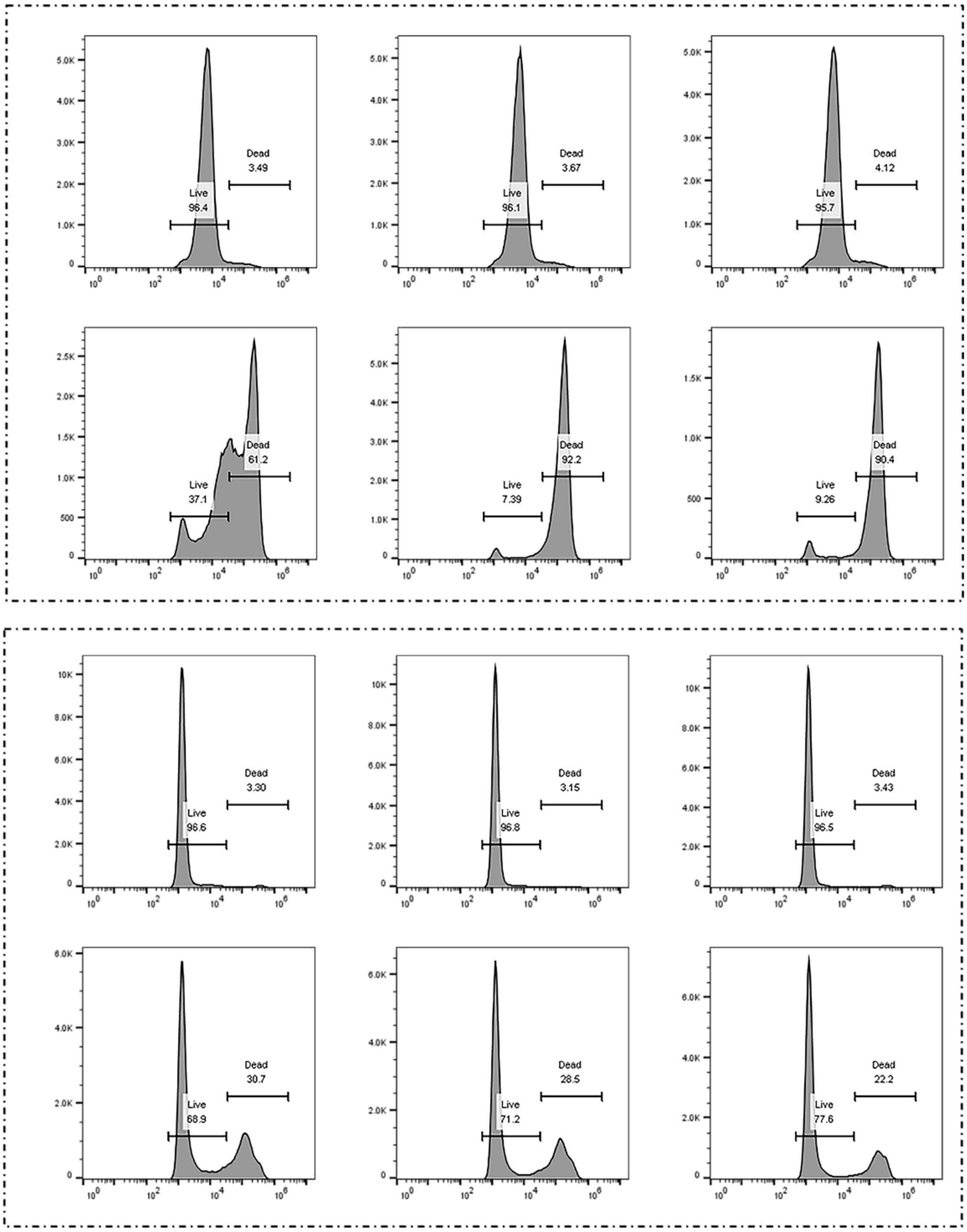


**Fig. S4.** Flow cytometry analysis of the death ratio of CAL27 and SCC7 disposed with or without *P. gingivalis* (MOI = 1000) for 24 hours. All experiments were independently repeated in triplicate.

**Fig. S5**

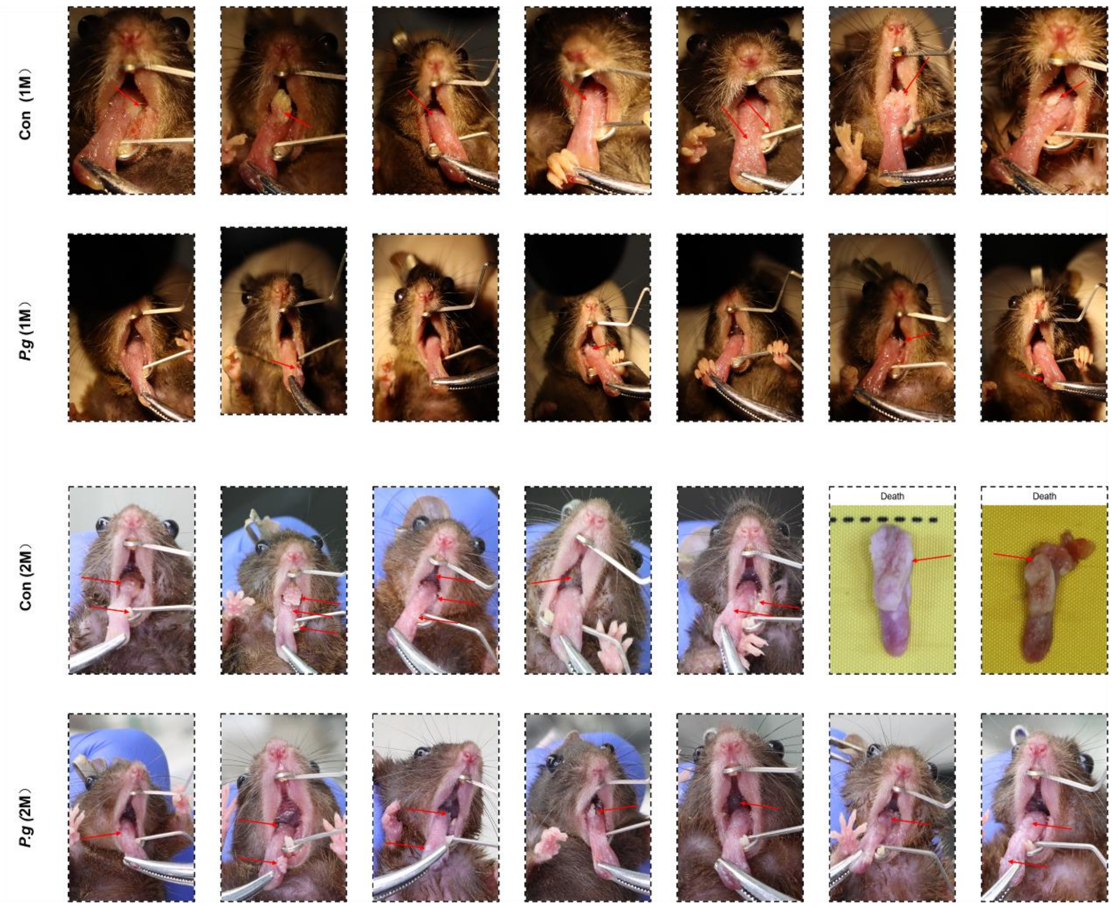


**Fig. S5.** Real-time monitoring tumors of 4NQO mice disposed with or without *P. gingivalis* for one month and two months (n=7).

**Fig S6**

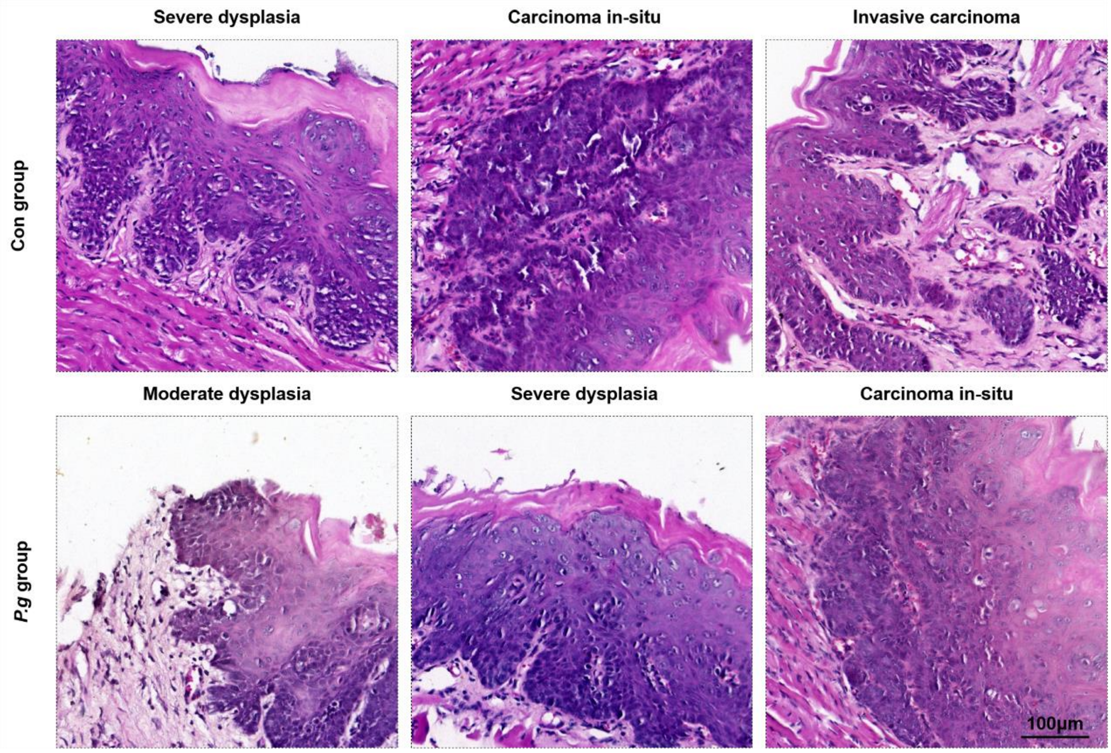


**Fig. S6** Representative pictures of HE staining, containing mild to severe dysplasia, carcinoma in situ and invasive carcinoma. (n=5, Scale Bar=100μm).

**Fig. S7**

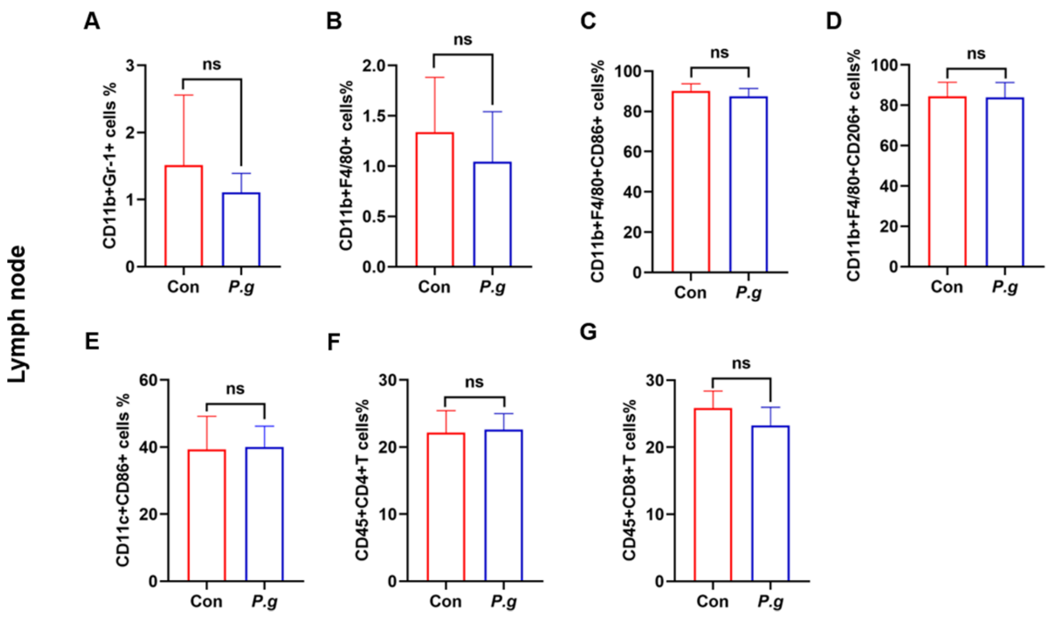


**Fig. S7.** Various infiltrating immune cell subsets in the lymph nodes of 4NQO-induced mice. The percentage of tumor-infiltrating **A.** CD11b$^+$ Gr-1$^+$ MDSCs, **B.** CD11b$^+$ F4/80$^+$ Macrophages, **C.** CD11b$^+$ F4/80$^+$ CD86$^+$ M1-like Macrophages, **D.** CD11b$^+$ F4/80$^+$ CD206$^+$ M2-like Macrophages, **E.** CD11c$^+$ CD86$^+$ Matured DCs, **F.** CD4$^+$ T cells and **G.** CD8$^+$ T cells were compared. All data are represented as Mean±SD, n=3. (Unpaired t test Welch's correction was used for MDSC, unpaired t test was used for the rest, ns, not significant).

**Fig. S8**

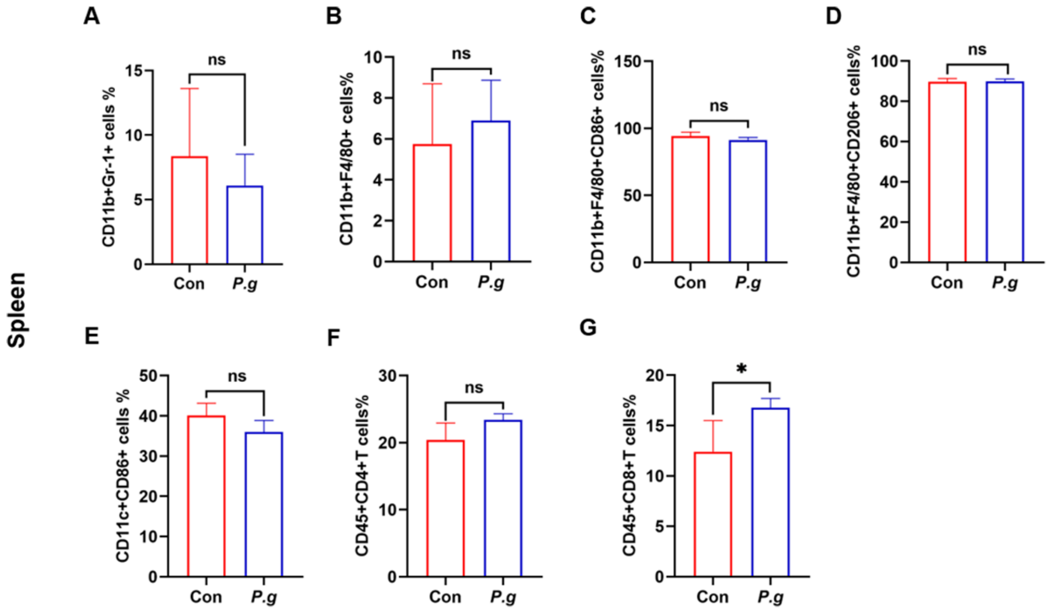


**Fig. S8.** Various infiltrating immune cell subsets in the spleen of 4NQO-induced mice. The percentage of tumor-infiltrating **A.** CD11b$^+$ Gr-1$^+$ MDSCs, **B.** CD11b$^+$ F4/80$^+$ Macrophages, **C.** CD11b$^+$ F4/80$^+$ CD86$^+$ M1-like Macrophages, **D.** CD11b$^+$ F4/80$^+$ CD206$^+$ M2-like Macrophages, **E.** CD11c$^+$ CD86$^+$ Matured DCs, **F.** CD4$^+$ T cells and **G.** CD8$^+$ T cells were compared. All data are represented as Mean±SD, n=3. (Unpaired t test with Welch's correction was used for CD8$^+$T cells, unpaired t test was used for the rest, *, $P < 0.05$; ns, not significant).
